# Evaluation of antinociceptive activity of *Ilex dipyrena Wall. in mice*

**DOI:** 10.1186/s12906-021-03357-4

**Published:** 2021-07-01

**Authors:** Amjad Ali, Abdul Nasir, Syed Wadood Ali Shah, Atif Ali Khan Khalil, Mi-jeong Ahn, Syed Muhammad Mukarram Shah, Fazli Subhan, Muhammad Faheem, Wasim Sajjad, Mohammad Shoaib, Saeed Ahmad, Nausheen Nazir, Mohammad Nisar

**Affiliations:** 1 Department of Botany, University of Malakand, Dir (Lower), Chakdara, Khyber Pakhtunkhwa 18800 Pakistan; 2 Department of Molecular Science and Technology, Ajou University, Suwon, 16499 Republic of Korea; 3 Department of Pharmacy, University of Malakand, Dir (Lower), Chakdara, Khyber Pakhtunkhwa 18800 Pakistan; 4 Department of Biological Sciences, National University of Medical Sciences, Rawalpindi, 46000 Pakistan; 5 College of Pharmacy and Research Institute of Pharmaceutical Sciences, Gyeongsang National University, Jinju, Gyeongsangnam-do 52828 South Korea; 6 Department of Zoology, University of Malakand, Dir (Lower), Chakdara, Khyber Pakhtunkhwa 18800 Pakistan; 7 Department of Biochemistry, University of Malakand, Dir (Lower), Chakdara, Khyber Pakhtunkhwa 18800 Pakistan

**Keywords:** *Ilex dipyrena*, Analgesic effect, Mouse models, Opioidergic, GABAergic

## Abstract

**Background:**

In order to find a new natural resource for pain-relief, the analgesic effects of *Ilex dipyrena* crude extract, fractions, and subfractions were evaluated in in-vivo mouse models with possible mechanism of action.

**Methods:**

Analgesic effects of crude extract (100 and 200 mg/kg body weight), fractions and subfractions (75 mg/kg body weight) were screened using heat-induced (tail-immersion and hot plate test) and chemical-induced (formalin and acetic acid) nociception models in mice. The samples were also tested for the elucidation of a possible mechanism through opioidergic and GABAergic systems.

**Results:**

The administration of crude extract, fractions and subfractions produced analgesic responses in acetic acid, formalin, tail immersion, and hot plate model for pain similar to those obtained with the standard. Naloxone antagonized the antinociceptive effects of the tested samples, whereas bicuculline showed partial inhibition. Considering the analgesic response, crude extract, fractions, and subfractions demonstrated promising inhibitory activity against all test models for pain, which was further supported by the possible involvement of opioidergic and GABAergic systems.

**Conclusion:**

The results suggest that this plant may be useful in the development of new analgesic drugs. Further research with regard to the isolation of bioactive compounds is required to verify these findings.

## Background

The use of medicinal plants for the treatment of ailments have a long history, gaining worldwide attention as a versatile source of therapeutic agents [1–3]. Pain, a distressing symptom of many diseases, has attracted substantial attention in the field of research, because of evoked adverse effects associated with available pain therapies [4–7]. Chronic pain affects about 50% of the global population and is a significant health concern because pain-creating mechanisms are not well understood, and adequate therapies are not available [8–10]. Pain is usually treated with the assistance of opioids or non-opioids analgesics, although commonly used medications for inflammation and pain such as non-steroidal anti-inflammatory drugs and corticosteroids carry multiple side effects [11]. For centuries, herbal medicines have been used because of their efficacy with minimal adverse effects and are the major source of countless useful compounds leading to novel drug development [12, 13]. Recent research has discovered active analgesics, aspirin, and morphine, from the medicinal plant with a substantial safety profile [14–16].

*Ilex* (Aquifoliaceae) comprising 400 species are found in tropical temperate regions. They are evergreen deciduous trees and shrubs found in India with almost 24 species [17]. Most of the species are used extensively for the various disease therapies in traditional herbal medicine worldwide due to the presence of saponins [18], flavonoids [19], aldehydes [20], hemiterpene glycosides, and triterpenes [21]. *Ilex latifolia* extracts have previously been reported to exhibit strong anti-inflammatory and antinociceptive effects in in-vitro and in-vivo models [22]. A purified saponin fraction derived from the root of *I. pubescens* showed significant analgesic effect in both visceral and central nociceptive models [23]. In addition, dried *I. paraguariensis* leaves and twigs (yerba mate), used in the preparation of a local tea known as “mate”, a commonly consumed beverage in many South American countries. Studies have shown that *I. paraguariensis* has many chemicals, including theobromine and caffeine, which are alkaloids with medicinal and pharmacological properties that can affect the central nervous system [24, 25]. Being an important member of this genus, *I. dipyrena* is an evergreen tree that reaches a height of approximately 10 m, is found abundantly in Pakistan and India [17]. The GC-MS analysis of fatty acids from *I. dipyrena* confirmed the presence of cembratriene, an active ingredient of several antitumor agents, and solanesol which is known to be used as antibiotic, cardiac stimulant and lipid antioxidant [26, 27]. The *I. dipyrena* leaf ethanolic extract displayed antimicrobial activity against *Escherichia coli*. However, there is no report on analgesic activity of this plant. Therefore, as our ongoing research to find a new natural resource for pain-relief, this study aimed to access the phenolic and flavonoid constituent of *I. dipyrena,* and their analgesic effect and possible mechanisms involved in authenticating its traditional use on scientific grounds. These preliminary findings will provide scientific base for the isolation and structural characterization of the plant bioactive molecules with improved analgesic and anti-inflammatory potential.

## Methods

### Collection and authentication of plant materials

The mature plant of *Ilex dipyrena* Wall. was collected from Shangla, Khyber-Pakhtunkhwa in July, 2018. The plant was identified by Professor Dr. Jehandar Shah, assigned the voucher number of BG/ID/18–244, and deposited at the College of Pharmacy, University of Malakand, Pakistan.

### Extraction

The whole plant (4.5 kg) was macerated in the 100% methanol, stirred occasionally for 10–15 days at 25 ± 2 °C, and filtered. The filtrate was concentrated using a rotary evaporator, resulting in 569 g of crude extract (Crd-Id), followed by fractionation with *n*-hexane (nhex-Id) 29 g, chloroform (Chl-Id) 63 g, ethyl acetate (Et-Id) 75 g, and butanol (But-Id) 27 g to obtain the respective fractions with remaining aqueous (Aq-Id) fraction 331 g [28].

### Fractionation and subfractionation

The pharmacologically active fraction (chloroform, Chl-Id, 63 g) was subjected to chromatography by a gravity silica-filled column. The elution began with *n*-hexane and gradually polarity was successively increased with ethyl acetate to attain 100% ethyl acetate. A total of 76 major fractions were collected (Fr. Chl-Id 1–9, Fr. Chl-Id 10–16, Fr. Chl-Id 17–22, Fr. Chl-Id 23–31, Fr. Chl-Id 32–43, Fr. Chl-Id 44–61 and Fr. Chl-Id 62–76) based on thin-layer chromatography (TLC) analysis and were visualized using vanillin-sulfuric acid reagent and iodine vapors. These fractions were then evaluated for bioactivity using analgesic activity test models.

### Preliminary phytochemical tests

The crude extract was subjected to qualitative chemical test for the identification of phytochemicals like flavonoids using sodium hydroxide and magnesium ribbon test, alkaloids using Dragendorff’s test, Keller Killanis test for glycosides, for saponins froth and emulsion test, for terpenoids chloroform and sulphuric acid test, for tannins ferric-chloride and gelatin test, for proteins xanthoproteic and Biuret, and for fats and oils filter paper tests were used as per reported methods [29, 30].

### Determination of total phenolic content

For the determination of the total phenolic contents, the Folin-Ciocalteu method was employed following the previously reported method [31]. Briefly, deionized water (0.5 mL) and Folin-Ciocalteu reagent (125 μL) were added to extract samples (125 μL). The reaction mixture was then incubated for 6 min followed by the addition of 1.25 mL (7%) aqueous sodium carbonate (Na_2_CO_3)_. After adding distilled water, the final volume was brought to 3 ml and incubated for 90 min, and the absorption spectra measured at 765 nm. The total phenolic contents were represented as gallic acid equivalents using the calibration curve (Gallic acid in mg/g of extract). Dilutions of 20, 40, 60, 80, and 100 mg/mL were used to construct a standard Gallic acid curve.

### Determination of total flavonoids

Flavonoids were determined using a technique based on a flavonoid-aluminum combination with a maximal absorption at 420 nm. Following that, a calibration curve was constructed using Quercetin. One milliliter of diluted sample was added with one milliliter of 2% aluminum trichloride (AICl_3_), methanolic solution, incubated for 15 mint at room temperature. Quercetin equivalents (QE quercetin in mg/g of extract) were employed as representative of total flavonoid content. A standard quercetin curve was constructed by preparing dilutions of 20, 40, 60, 80, and 100 mg/mL [31].

### Chemicals

Diclofenac sodium and tramadol were gifted by Alliance Pharmaceuticals and Aries Pharmaceuticals, Peshawar, KPK, Pakistan. Silica, TLC plates, and bicuculline were purchased from Sigma-Aldrich, Germany. Methanol, chloroform, hexane, ethyl acetate, butanol, naloxone, acetic acid and formalin were bought from Merck (Darmstadt, Germany).

### Animals and ethical approval

Male Balb/C mice of 19–24 g body weight (b.w.) were obtained from the National Institute of Health, Islamabad, Pakistan, and quarantined in an animal house under standard laboratory conditions (25 ± 2 °C and 55–65% relative humidity and 12 h light/12 h dark cycle) with a standard diet and water provided ad libitum. In addition, after experimentation, the animals were sacrificed by euthanasia with isoflurane. All protocols used in the study were approved by the Departmental Ethical Committee of the University (Pharm/EC-Id/37–12/18) in accordance with the Animal Bye-Laws 2008, Scientific Procedures Issue-I of the University of Malakand.

### Animal grouping and dosing

The experimental animals were randomly divided into a number of groups (control, test groups, and standard) comprising of eight animals each. The control group received 2% Tween 80 as the vehicle. The test groups were given a dose of 100 and 200 mg/kg b.w for crude extract (Crd-Id), while 75 mg/kg b.w for fractions (Chl-Id and Et-Id) and subfractions (Fr.Chl-Id 46–48) to the respective group. The standard group was administered with standard diclofenac sodium (10 mg/kg b.w.) for acetic acid-induced writhing, indomethacin (10 mg/kg b.w.) and morphine (5 mg/kg b.w.) for formalin induce licking response, tramadol (20 mg/kg b.w.) for tail immersion, and hot plate model respectively. In addition, the selection of doses of crude extract, fractions, subfractions was based on the preliminary pharmacological activity.

### Acute toxicity

The acute toxicity of crude extract, fractions, and subfractions of *I. dipyrena* was investigated following the standard protocol [32, 33]. Different dose concentrations such as 100, 250, 500, 1000, 1500, and 2000 mg/kg were administered orally in various groups of experimental mice (*n* = 8). For negative control one group was given normal saline. Each group of mice was examined for 24 h for any adverse effects or mortality, followed by 14 days with free access to water and food. Animals were observed daily for 2 weeks to observe signs of convulsions, tremor, diarrhea, salivation, lethargy, and sleeping. The body weight was also measured as per weekly observation.

### Analgesic activity

#### Acetic acid-induced writhing test

The mice in the experimental groups (*n* = 8) received crude extract, fractions and subfractions (i.p.) at various dose concentrations (100 and 200 mg/kg b.w for crude extract while 75 mg/kg b.w for fractions and subfractions) 30 min prior to acetic acid administration (0.6%, 10 mL/kg, i.p.). The negative control group received 10 mL/kg of 1% solution of Tween 80 (1%, v/v) and the positive control group received 10 mg/kg (i.p.) of diclofenac sodium. The intensity of nociception was recorded in the number of writhes produced within 30 min of acetic acid administration [34].

#### Formalin test

The experimental mice groups (*n* = 8) received crude extract, fractions, and subfractions (i.p.) at different dose concentrations 1 h prior the treatment of animals in the respective groups were treated with formalin (1%, 50 μL) on the right hind paw. The treated paw of mice was observed through a plexiglass box for 30 min and the paw licking of mice was recorded in seconds in two phases, 0–5 min (neurogenic pain), and 15–30 min (inflammatory pain) [35].

#### Tail immersion test

The test was conducted according to a previously published protocol, which involved measuring the time required for mice to flick their tails away from hot water maintained at 55 ± 2 °C. After administration of the crude extract, fractions, and subfractions or vehicle (10 mL/kg, i.p.) the data were recorded for 30 min. Approximately 20 mg/kg dose of tramadol was given by subcutaneous (s.c.) route for 30 min before the tail immersion test [36].

#### Hot plate test and involvement of opioid system

This method was used to investigate the latency response of mice after administration of the crude extract, fractions, subfractions, and standard. Each mouse in each group was placed on a hot plate (50 ± 2 °C) once the sample was administered. The latency was measured in seconds for licking, shaking the paw, or jumping off the hot surface, with a 60-s cutoff time [37].

#### Investigations on the mechanism of action

For the possible involvement of the opioid system, naloxone hydrochloride (opioid receptor antagonist, 2 mg/kg, i.p.) was injected 15 min before the administration of either tramadol or crude extract, fractions, and subfractions. The hot plate test was applied to record the response latencies at time intervals of 0, 30, 60, 90, and 120 min. The same 20 s cut-off time was used for ensuring the animals’ safety [36, 38].

To assess the possible participation of the γ-aminobutyric acid (GABAergic) pathway, the mice were pretreated via the i.p. route with bicuculline, (1.0 mg/kg), and after 15 min, they received crude extract, fractions, and subfractions. The pain produced by acetic acid was analyzed 30 min after the administration of bicuculline a positive control, and crude extract, fractions, and subfractions [39].

### Statistical analysis

The data were represented as mean ± SEM (standard error of the mean). For statistical analysis, one-way ANOVA followed by the Dunnett’s test was carried out with GraphPad Prism 5 version 5.01 (GraphPad Prism Software, Inc., SanDiego, CA, USA). The results were considered significant at *P* < 0.05. The results of tail immersion and hot plate tests were calculated with the following formula and were represented as the percentage of the maximal possible effect (%MPE):

$$ \mathrm{MPE}\ \left(\%\right)=\left[\left(\mathrm{postdrug}\ \mathrm{latency}\right)-\left(\mathrm{predrug}\ \mathrm{latency}\right)/\left(\mathrm{cutoff}\ \mathrm{time}\right)-\left(\mathrm{predrug}\ \mathrm{latency}\right)\right]\times 100 $$

## Results

### Phytochemical screening

The results of the phytochemical screening of crude extract (Crd-Id) are summarized in Table 1. The presence of tannins, saponins alkaloids, glycosides, flavonoids, phenolics, and terpenoids were revealed.

**Table 1:** Phytochemical screening of crude extract of *I. dyperena*

Phytochemicals	Test Performed	Results
Tannins	Ferric-chloride/ Gelatin	+/+
Saponins	Froth/Emulsion	+++/+++
Alkaloids	Dragendorff’s	+
Glycosides	Keller Killanis	+
Flavonoids	NaOH/Mg ribbon	+/+
Phenolics	Ferric-chloride	+++
Terpenoids	Chloroform, sulphuric acid	+++
Proteins	Xanthoproteic/Biuret	+/+
Fats and oils	Filter paper	+

Notes: +++: Strong positive test; +: Weak positive test

### Total phenolic and total flavonoid contents

Results of total phenolic and total flavonoid contents in the crude extract and fractions of *I. dipyrena* are presented in Table 2. Results showed that the chloroform and ethyl acetate fractions exhibited the highest phenolic contents with the mean values of 57.69 ± 0.73 and 45.91 ± 0.93, respectively, of Gallic acid equivalent per gram (mg GAE/g) of the dry sample (Table 2).

**Table 2:** Total phenolic and flavonoid of crude extract, fractions and subfractions of *I. dipyrena*

Sample	TPC (mg GAE/g)	TFC (mg QE/g)
Crd-Id	31.22 ± 0.39	39.84 ± 0.81
Hex-Id	26.54 ± 0.91	31.33 ± 1.04
Chl-Id	57.69 ± 0.73	73.55 ± 0.91
Et-Id	45.91 ± 0.93	64.39 ± 0.83
But-Id	41.62 ± 1.07	49.21 ± 1.01
Aq-Id	19.75 ± 0.87	28.16 ± 0.91

All values are expressed as mean ± SEM, *n* = 3, *TPC* Total phenolic contents, *TFC* Total flavonoid, *Crd-Id* Crude extract, *Hex-Id* N-hexane fraction, *Chl-Id* Chloroform fraction, *Et-Id* Ethyl acetate fraction, *But-Id* Butanol fraction, *Aq-Id* Aqueous fraction

Results of total flavonoid contents revealed that the chloroform and ethyl acetate fractions exhibited the highest flavonoid contents with the mean values of 73.55 ± 0.91 and 64.39 ± 0.83 mg of Quercetin equivalent per gram (mg QE/gm) of dry sample respectively (Table 2). Results displayed that chloroform and ethyl acetate fractions showed the highest flavonoid contents and total phenolic content. In contrast, the hexane and aqueous fractions showed the lowest phenolic and flavonoid content.

### Acute toxicity test

The crude extract (Crd-Id), fractions (Chl-Id and Et-Id), and subfractions (Fr.Chl-Id 46–48) revealed no mortality even at a maximum dose up to 1750 mg/kg (b.w) when administered orally. Animals were observed daily for 2 weeks and no signs of convulsions, tremor, diarrhea, salivation, lethargy, and sleeping was observed. The body weight was also normal as per weekly observation. Hence, 100 and 200 mg/kg dose for crude extract and 75 mg/kg for subsequent fractions and subfractions were chosen for the evaluation of pharmacological activities followed by preliminary pharmacological screening to confirm the doses.

### Acetic acid-induced writhing test

**Fig. 1:**
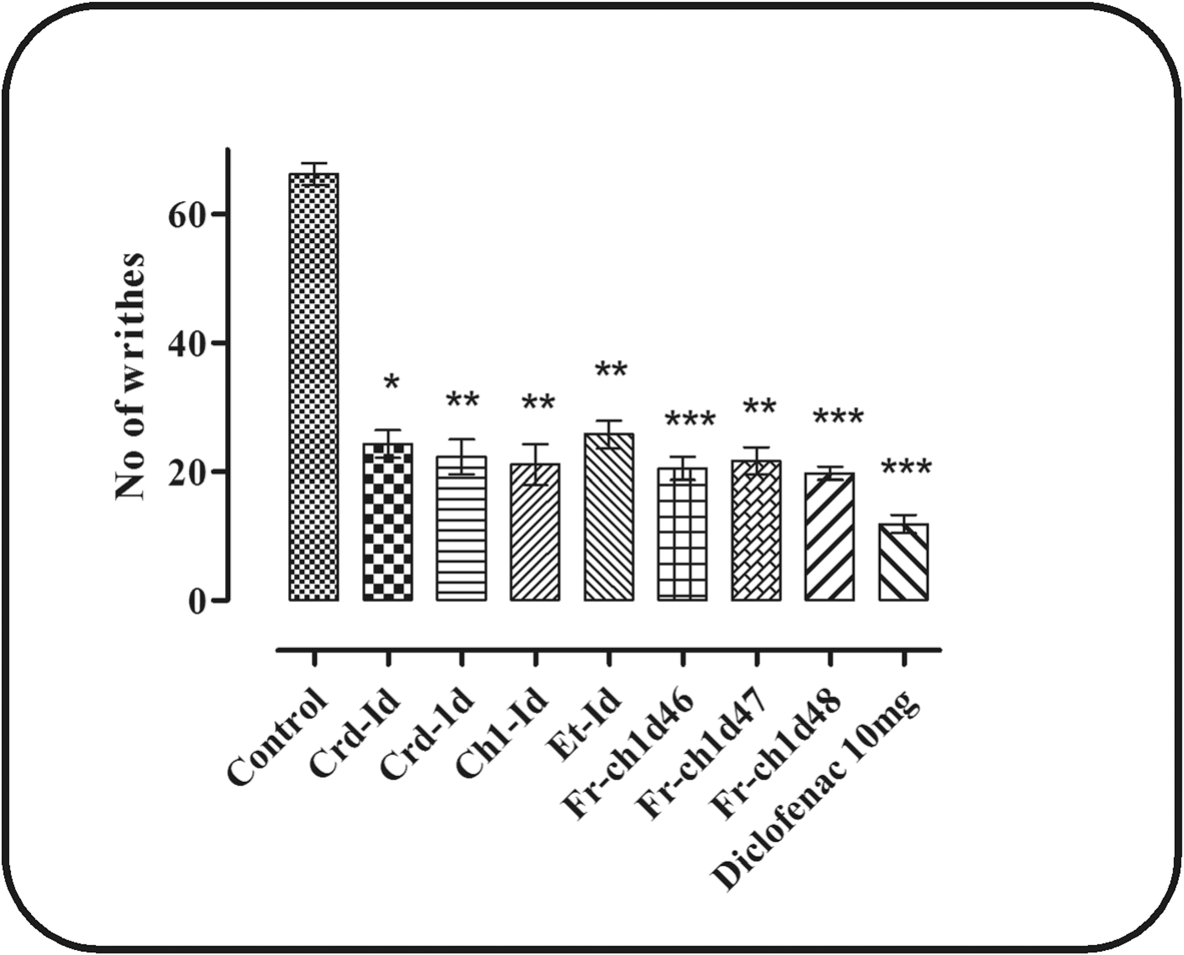
analgesic activity of *I. dipyrena* using acetic acid model. Mean ± SEM (*n* = 8). Where, ^*^P < 0.05, ^**^P < 0.01 and ^***^P < 0.001 statistically significant relative to control

While investigating the antinociceptive effect through the acetic acid-induced writhing test, the test samples exhibited a considerable analgesic activity. The data analysis of one-way ANOVA followed by Tukey’s multiple comparison test showed that crude extract (Crd-Id) at a dose of 100 and 200 mg/kg b.w inhibited the acetic acid induced writhing with a value of 63.29%, (*P* < 0.05) and 66.33% (*P* < 0.01). Apart from Crd-Id, the chloroform (Chl-Id) and ethyl acetate (Et-Id) fractions at a low dose of 75 mg/kg, produced 68.14 (*P* < 0.01) and 61.36% (*P* < 0.001) responses respectively, as shown in Fig. 1 and Table 3.

**Table 3:** Acetic acid-induced analgesic activity of the crude extract, fractions, and subfractions of *I. dipyrena*

Treatment	Dose	Number of writhing	% inhibition
Control	(2% Tween 80)	66.19 ± 1.71	–
Crd-Id	100	24.29 ± 2.15^*^	63.29
Crd-Id	200	22.28 ± 2.71^**^	66.33
Chl-Id	75	21.11 ± 3.19^**^	68.14
Et-Id	75	25.57 ± 2.18^**^	61.36
Fr.Chl-Id 46	75	20.48 ± 1.78^***^	69.03
Fr.Chl-Id 47	75	21.63 ± 2.12^**^	67.31
Fr.Chl-Id 48	75	19.73 ± 1.06^***^	70.19
Diclofenac	10	11.89 ± 1.41^***^	82.03

Each value is represented as Mean ± SEM (*n* = 8). Where, ^*^P < 0.05, ^**^P < 0.01 and ^***^P < 0.001 statistically significant relative to control. *Crd-Id* Crude extract, *Hex-Id* n-hexane fraction, *Chl-Id* Chloroform fraction; *Et-Id* Ethyl acetate fraction, *Fr.Chl-Id 46, 47 and 48* subfractions

Whereas, the hexane, butanol, and aqueous fraction displayed no considerable effects (data not shown). In addition, from the pharmacologically active chloroform fraction Chl-Id, the inhibitory effect of subfractions Fr.Chl-Id 46, 47, and 48 (75 mg/kg) were also observed with the maximum value of 69.03% (*P* < 0.001), 67.31% (*P* < 0.01) and 70.19%, (*P* < 0.001) respectively and has almost similar inhibitory effect compared to diclofenac sodium (82.03%, *P* < 0.001). The one way ANOVA followed by Tukey’s multiple comparison test revealed that the effects of chloroform (Chl-Id), ethyl acetate (Et-Id) fractions, subfractions Fr.Chl-Id 46, 47 and 48 were similar to crude extract (Crd-Id) and standard diclofenac sodium, without statistically significant differences.

### Formalin test

**Fig. 2:**
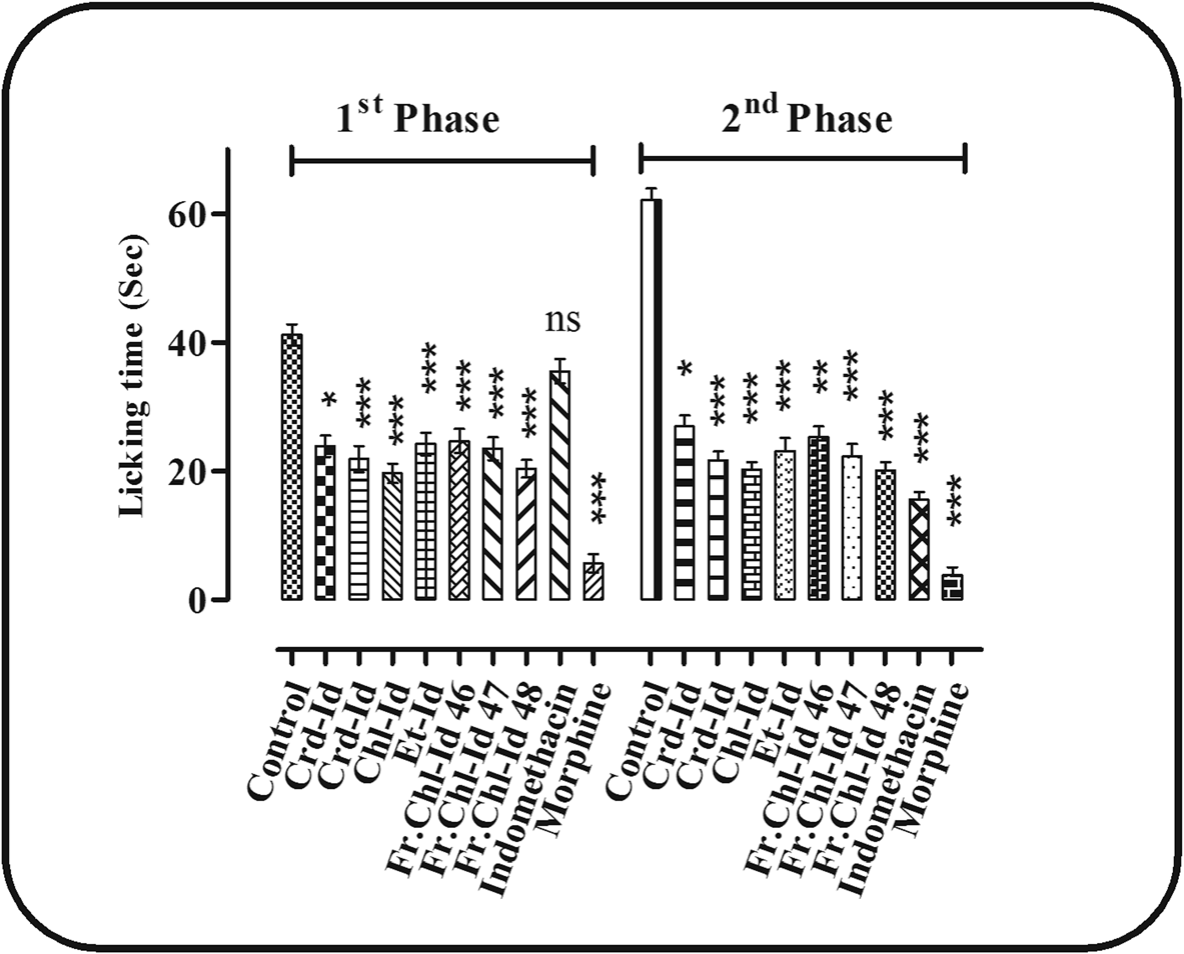
Formalin-induced licking response in 1st and 2nd Phase. Mean ± SEM (*n* = 8). Where, ^*^P < 0.05, ^**^P < 0.01 and ^***^P < 0.001 statistically significant relative to control

An inhibitory effect was produced by crude extract, fractions, and subfractions in formalin induced nociception by considerably impeding both the neurogenic and inflammatory phases of the licking test. In 1st phase, data analysis using one way ANOVA followed by Tukey’s multiple comparison test revealed that crude extract (Crd-Id) at a dose of 100 and 200 mg/kg b.w, chloroform (Chl-Id), ethyl acetate (Et-Id) fractions, subfractions Fr.Chl-Id 46, 47 and 48 at a dose of 75 mg/kg b.w significantly reduced neurogenic pain by 42.06, 46.99, 52.29, 41.32, 40.12, 43.07 and 50.59%, respectively, compared to control, while indomethacin did not induce a significant reduction of neurogenic pain. Morphine produced a significant response in 1st phase (Fig. 2).

In the 2nd phase, the test samples and standard treatments significantly reduced inflammatory pain by 56.58, 65.11, 67.41, 63.29, 59.21, 64.12 67.57, 74.81, and 93.78%, respectively, compared to control (Table 4).

**Table 4:** Effects of the crude extract, fractions, and subfractions of *I. dipyrena* on formalininduced paw-licking response

Treatment	Dose	Licking time (Sec)		Inhibition (%)	
		1st Phase	2nd Phase	1st Phase	2nd Phase
Control	2% Tween-80	41.22 ± 1.68	62.11 ± 1.86	–	–
Crd-Id	100	23.88 ± 1.65^*^	26.96 ± 1.74^*^	42.06	56.58
Crd-Id	200	21.85 ± 2.05^***^	21.66 ± 1.38^***^	46.99	65.11
Chl-Id	75	19.66 ± 1.48^***^	20.24 ± 1.21^***^	52.29	67.41
Et-Id	75	24.18 ± 1.76^***^	23.13 ± 1.99^***^	41.32	63.29
Fr.Chl-Id 46	75	24.68 ± 1.86^**^	25.33 ± 1.61^**^	40.12	59.21
Fr.Chl-Id 47	75	23.46 ± 1.81^***^	22.28 ± 1.91^***^	43.07	64.12
Fr.Chl-Id 48	75	20.36 ± 1.38^***^	20.14 ± 1.22^***^	50.59	67.57
Indomethacin	10 mg	35.58 ± 1.92^ns^	15.57 ± 1.25^***^	13.68	74.81
Morphine	5 mg	5.66 ± 1.42^***^	3.85 ± 1.22^***^	74.06	93.78

Each value is represented as Mean ± SEM (*n* = 8). Where, ^*^P < 0.05, ^**^P < 0.01 and ^***^P < 0.001 statistically significant relative to control. *Crd-Id* Crude extract, *Hex-Id* n-hexane fraction, *Chl-Id* Chloroform fraction, *Et-Id* Ethyl acetate fraction, *Fr.Chl-Id 46, 47 and 48* subfractions

The data analysis of one way ANOVA followed by Tukey’s multiple comparison test showed that the effects of chloroform (Chl-Id), ethyl acetate (Et-Id) fractions, subfractions Fr.Chl-Id 46, 47, and 48 were similar to crude extract (Crd-Id) and standard treatments, without statistically significant differences (Fig. 2). In addition, other fractions like *n*-hexane displayed no promising response (data not shown).

### Tail immersion test

The tail immersion test is frequently used to determine the samples’ central analgesic effect. Inhibition response in the form of latency response in seconds and the percentage was shown by crude extract, fractions, and subfractions as compared to the control group (*P* < 0.01 and *P* < 0.001). The data analysis of one-way ANOVA followed by Tukey’s multiple comparison test showed that the crude extract (Crd-Id) at a dose of 100 and 200 mg/kg b.w produced latency response with a value of 57.15%, (8.89 ± 1.73, *P* < 0.001) and 60.35% (9.43 ± 1.03, *P* < 0.001). The chloroform (Chl-Id) and ethyl acetate (Et-Id) fractions at a low dose of 75 mg/kg b.w, produced 62.72 (10.25 ± 1.49, *P* < 0.01) and 58.71% (9.49 ± 1.82, *P* < 0.001) response respectively, as shown in Table 5. Tramadol, a positive control demonstrated 75.98% (15.86 ± 1.17, *P* < 0.001) inhibitory effect at a therapeutic dose.

**Table 5:** Effects of the crude extract, fractions, and subfractions of *I. dipyrena* on tail immersion analgesic response

Treatment	Dose	Pre Treatment	Post Treatment	% inhibition
Control (2% Tween 80)	–	3.97 ± 2.09	4.09 ± 1.21	–
Crd-Id	100	3.81 ± 2.09	8.89 ± 1.73^***^	57.15
Crd-Id	200	3.74	9.43 ± 1.03^***^	60.35
Chl-Id	75	3.82 ± 1.81	10.25 ± 1.49^**^	62.72
Et-Id	75	3.92 ± 1.67	9.49 ± 1.82^**^	58.71
Fr. Chl-Id 46	75	4.01 ± 1.39	10.06 ± 1.37^**^	60.16
Fr. Chl-Id 47	75	3.94 ± 1.67	10.68 ± 1.52^**^	63.12
Fr. Chl-Id 48	75	3.75 ± 1.92	11.50 ± 1.19^***^	67.39
Tramadol	20 mg	3.81 ± 1.81	15.86 ± 1.17^***^	75.98

Each value is represented as Mean ± SEM (*n* = 8). Where, ^*^P < 0.05, ^**^P < 0.01 and ^***^P < 0.001 statistically significant relative to control. *Crd-Id* Crude extract, *Hex-Id* n-hexane fraction, *Chl-Id* chloroform fraction, *Et-Id* Ethyl acetate fraction, *Fr.Chl-Id 46, 47 and 48* subfractions

The maximum percent inhibition of subfractions Fr.Chl-Id 46, 47 and 48 showed 60.16% (10.06 ± 1.37, *P* < 0.001), 63.12% (10.68 ± 1.52, *P* < 0.01) and 67.39% (11.50 ± 1.19, *P* < 0.001, *n* = 8) respectively at 75 mg/kg b.w for each sub-fraction (Table 5).

### Hot plate test and involvement of opioid system

As shown in Table 6, the analgesic potential of crude extract (Crd-Id) at a dose of 100 and 200 mg/kg b.w, chloroform (Chl-Id), ethyl acetate (Et-Id) fractions, subfractions Fr.Chl-Id 46, 47 and 48 at a dose of 75 mg/kg in hot plate test and involvement of opioid system. The analgesic effect of crude extract, fractions, and subfractions was dose reliant and significant at 60 min for samples. At 60 min, the maximum effects in the form latency increased were observed to 7.79 ± 1.10 (57.17%, *P* < 0.001)_,_ 8.54 ± 1.10 (63.11%, *P* < 0.001), 9.61 ± 2.31 (67.22%, *P* < 0.01) and 8.07 ± 1.62 (60.96%, *P* < 0.001) respectively for Crd-Id, Chl-Id, and Et-Id.

**Table 6:** Effects of crude extract, fractions and subfractions of *I. dipyrena* on hot plate and involvement of opioid system

Treatment	Doses	0 min	30 min	60 min	90 min	120 min	At 60 min
Control	–	3.19 ± 1.57	3.07 ± 2.01	3.15 ± 1.12	3.09 ± 1.21	3.11 ± 2.14	–
Crd-Id	100	3.29 ± 2.15	5.31 ± 1.11	7.79 ± 1.10^***^	7.11 ± 1.91	6.69 ± 1.74	57.15%
Crd-Id	200	3.40 ± 1.51	5.77 ± 1.46	8.54 ± 1.10^***^	8.02 ± 1.91	7.21 ± 2.31	63.11%
Chl- Id	75	3.12 ± 2.31	6.31 ± 1.91	9.61 ± 2.31^**^	9.13 ± 1.51	8.11 ± 1.46	67.22%
Et- Id	75	3.21 ± 1.63	6.41 ± 1.47	8.07 ± 1.62^***^	9.72 ± 1.35	8.19 ± 2.71	60.96%
Fr.Chl-Id 46	75	3.61 ± 1.73	6.32 ± 1.71	8.19 ± 1.38^***^	9.18 ± 1.53	8.27 ± 2.11	61.53%
Fr.Chl-Id 47	75	3.28 ± 2.05	5.49 ± 1.91	8.87 ± 1.09^***^	7.59 ± 1.56	6.58 ± 1.47	64.48%
Fr.Chl-Id 48	75	3.13 ± 2.11	6.27 ± 1.31	9.87 ± 1.47^**^	10.21 ± 2.17	9.34 ± 1.49	68.08%
Tramadol	20	3.01 ± 1.18	7.71 ± 1.21	17.01 ± 1.91^***^	12.19 ± 1.37	11.21 ± 1.67	81.48%
Crd- Id +Naloxone	100 + 2	3.09 ± 1.14	3.07 ± 1.59	3.32 ± 2.12	3.29 ± 1.91	3.01 ± 1.91	–
Crd- Id +Naloxone	200 + 2	3.02 ± 1.41	3.11 ± 1.91	3.22 ± 1.67	3.31 ± 1.83	3.12 ± 1.67	–
Chl- Id +Naloxone	75 + 2	3.01 ± 2.21	3.16 ± 1.16	3.41 ± 1.87	3.11 ± 2.31	3.12 ± 1.29	–
Et- Id +Naloxone	75 + 2	3.03 ± 1.51	3.42 ± 1.13	3.11 ± 1.28	3.57 ± 1.72	3.16 ± 2.01	–
Fr.Chl-Id 46 + Naloxone	75 + 2	3.11 ± 1.22	3.33 ± 1.87	3.37 ± 1.76	3.41 ± 1.24	3.30 ± 1.19	–
Fr.Chl-Id 47 + Naloxone	75 + 2	3.38 ± 1.67	3.52 ± 1.91	3.56 ± 1.31	3.31 ± 1.71	3.49 ± 1.87	
Fr.Chl-Id 48 + Naloxone	75 + 2	3.37 ± 1.91	3.18 ± 1.28	3.21 ± 1.93	3.42 ± 1.37	3.11 ± 2.03	–
Tramadol+Naloxone	20 + 2	3.21 ± 1.31	3.17 ± 2.11	3.41 ± 1.61	3.29 ± 1.92	3.17 ± 1.16	–

Each value is represented as Mean ± SEM (*n* = 8). Where, ^*^P < 0.05, ^**^P < 0.01 and ^***^P < 0.001 statistically significant relative to control. *Crd-Id* Crude extract, *Hex-Id* n-hexane fraction, *Chl-Id* chloroform fraction, *Et-Id* Ethyl acetate fraction, *Fr.Chl-Id 46, 47 and 48* subfractions

The subfractions (Fr.Chl-Id 46–48) from Chl-Id showed maximum latency responses of 8.19 ± 1.38 (61.53%, *P* < 0.001)_,_ 8.87 ± 1.09 (64.48%, *P* < 0.001) and 9.87 ± 1.47 (68.08%, *P* < 0.01) respectively. Tramadol exhibited effect over the experimental session and powerful activity was recorded at 60 min after treatment 81.66%, *P* < 0.001. The effects of crude extract (Crd-Id), chloroform (Chl-Id), ethyl acetate (Et-Id) fractions, subfractions Fr.Chl-Id 46, 47, and 48 were similar to standard Tramadol, without statistically significant differences after applying one way ANOVA followed by Tukey’s multiple comparison test. Animals pretreated with naloxone displayed a significant reduction in the activity of tramadol and tested samples (Table 6). To evaluate the participation of the opioid receptor in the analgesic effect of crude extract, fractions, and subfractions, naloxone was administered. The analgesic effect of Tramadol was completely antagonized representing the confirmation of the experiments. Analgesic effect induced by crude extract (100 and 200 mg/kg), fractions, and subfractions (75 mg/kg) was completely reserved in groups treated with naloxone attesting the participation of the opioid receptor.

### Investigation of GABAergic system

The crude extract, fractions, and subfractions displayed significant effect in protecting the mice constriction stimulated by acetic acid in the absence of bicuculline (Table 7). However, the co-administration of bicuculline reduced the pain response of the tested samples in mice in the acetic acid-induced pain model that was different from the samples when tested alone. The co-administration of crude Crd-Id at a dose of 100 and 200 mg/kg with bicuculline significantly reduced the pain response to 26.70 and 31.92%, respectively, which was significantly lower than that of crude Crd-Id (63.29 and 66.33%, Table 7) alone. The similar results were observed by fractions (Chl-Id and Et-Id) and subfractions (Fr.Chl-Id 46–48). These results suggest that GABA receptor blockade by bicuculline diminish the analgesic effects of crude extract, fractions, and subfractions. As a result, the antinociceptive effect of crude extract or fractions may be mediated via GABA_A_ receptors. GABA’s widespread distribution in the peripheral and central nervous systems established its role in the transmission and perception of pain impulses. Pre-treatment with bicuculline significantly decreased the analgesic effect of crude extract, fractions, and subfractions and the partial reversal was observed.

**Table 7:** Effect of GABA_A_ antagonist bicuculline on the analgesic response of samples

Treatment Dose		Without Bicuculline		With Bicuculline	
		Writhing count	Inhibition (%)	Writhing count	Inhibition (%)
Control (2% Tween 80)		66.19 ± 1.71	–	64.44 ± 1.83	–
Crd-Id	100	24.29 ± 2.15^*^	63.29	47.23 ± 2.11^ns^	26.70
Crd-Id	200	22.28 ± 2.71^**^	66.33	43.87 ± 1.66^*^	31.92
Chl-Id	75	21.11 ± 3.19^**^	68.14	42.22 ± 0.98^*^	34.48
Et-Id	75	25.57 ± 2.18^**^	61.36	49.39 ± 1.78^ns^	23.35
Fr.Chl-Id 46	75	20.48 ± 1.78^***^	69.03	48.48 ± 2.01^ns^	24.76
Fr.Chl-Id 47	75	21.63 ± 2.12^**^	67.31	47.31 ± 1.77^ns^	26.58
Fr.Chl-Id 48	75	19.73 ± 1.06^***^	70.19	41.19 ± 1.91^*^	36.08

Each value is represented as Mean ± SEM (*n* = 8). Where, ^*^P < 0.05, ^**^P < 0.01 and ^***^P < 0.001 statistically significant relative to control, (ns) not significant. *Crd-Id* Crude extract, *Hex-Id* n-hexane fraction, *Chl-Id* chloroform fraction, *Et-Id* Ethyl acetate fraction, *Fr.Chl-Id 46, 47 and 48* subfractions

## Discussion

Phytochemicals are naturally occurring secondary metabolites in plants and exhibit defensive, protective, and curative potential. Their regular intake as dietary sources may promote healthy life by protecting against various diseases [40]. Preliminary phytochemical screening of *I. dipyrena* revealed the presence of phytoconstituents such as glycosides, alkaloids, steroids, tannins, saponins, flavonoids, terpenoids, proteins, fats, and carbohydrates, and was consistent with the results obtained for other species of *Ilex* [21]. Our results on phytochemical screening displayed the presence of maximum phytoconstituents like glycosides, alkaloids, steroids, tannins, saponins, flavonoids, terpenoids, protein, fats, and carbohydrates, and was consistent with the results of other species [41]. The presence of a variety of secondary metabolites in the various samples of *I. dipyrena* is thought to be involved in its diverse pharmacological potential, including the analgesic effect following various pain receptors.

The presumed analgesic activity of the crude extract, fractions, and subfractions of *I. dipyrena* to elucidate the pain-relieving effects were also investigated. A number of analgesic testing methodologies such as acetic acid, formalin, tail immersion, and hot plate tests were employed to identify possible peripheral and central effects. To study the peripheral analgesic effects of drugs, the acetic-acid writhing test is usually used. The writhing test induces a painful sensation of peripheral origin in mice by administering irritants like acetic acid and phenylquinone. The analgesic activity is inferred from the decrease in the frequency of writhing. Sigmund et al. reported that abdominal writhing in mice was characterized by an arching of the back, contraction of the abdominal musculature, and extension of hind limbs [42]. However, the test is not specific as anticholinergic, antihistaminic, and other agents also display their activity in the test, thus, it is employed broadly for the screening of analgesic effects [43]. In our study, we found that the crude extract, fractions, and subfractions (200, 100, and 75 mg/kg b.w, respectively) exhibited antinociceptive effects in the acetic acid-induced writhing response (Table 3). This can be attributed to the inhibitory effects of the tested samples against the synthesis of arachidonic acid metabolites [44].

The in vivo formalin-induced paw pain model is well recognized as a valid standard for analgesic study [45]. The formalin test results in a separate biphasic response and in the early and late phases of this test, different analgesics can act differently. Therefore, the proposed method can be used to understand the mechanism of the anti-nociceptive effect. Drugs that act centrally, such as opioids, inhibit both phases, whereas drugs that act peripherally, such as indomethacin, dexamethasone, and aspirin, inhibit only the late phase [43]. The crude extract, fractions, and subfractions significantly (*P* < 0.01 and *P* < 0.001) (Table 4) decreased the formalin-induced nociceptive responses of both phases, however, their effects were highly pronounced in the inflammatory phase, thus suggesting the possible involvement of supraspinal systems in the analgesic response. These findings indicate that the *I. dipyrena* has both central analgesic and anti-inflammatory properties.

Naloxone at the given concentration also diminished the morphine-induced latency time in both experimental models. In the current study, we can see that the activity of tramadol (standard opioid analgesic) and that of the test samples proceeded parallel and are almost comparable. Similarly, the analgesic potential of test samples and tramadol was considerably affected by the prior administration of naloxone. This clearly shows the involvement of opioid receptors, which have been blocked by naloxone and the test samples, and tramadol was unable to exhibit analgesic response via the opioid receptors. In the nociception-mediated neuronal pathway, a vital role is played by the inhibitory GABAergic system at many sites. The pain signals from the periphery to the higher central nervous area are regulated by GABA_A_ receptors that are found in the dorsal horn of the spinal cord. Reducing the GABA inhibitory potential at spinal cord region is an indication of various painful conditions [46]. Various GABA receptor agonists like 4,5,6,7-tetra hydro isoxazole (5,4) pyridine 3-ol (THIP) and muscimol have shown antinociception via GABAergic system [47]. The analgesic effect of crude extract, fractions, and subfractions in the current study was partly reversed by bicuculline pretreatment. This result imply that the test samples could utilize GABAergic mechanism in mediating their analgesic effect. The involvement of GABA_A_ receptors in the modulation of pain by the test samples could be clearly understood in the current study. Bicuculline pretreatment blocks GABA_A_ receptors, and after blocking these receptors, the administration of test samples showed a partial analgesic effect similar to that of the analgesic effect in the absence of bicuculline. Therefore, it could be inferred that a part of the analgesic effect of the crude extract, fractions, and subfractions of *I. dipyrena* is because of the involvement of GABA_A_ receptors in the test animals.

In a recent report, over 15,000 flavonoids with assorted pharmacological effects have been cited [48]. Some analgesic flavonoids are promising in new analgesics drug development and attracted great interest from a large number of academic researchers and advanced users [49].

Taken together, the current study attested to the role of *I. dipyrena* as an analgesic, this species could serve as a promising candidate and warrants particular consideration in further research and development for the management of pain.

## Conclusions

The crude extract, fractions, and subfractions of *I. dipyrena* showed significant analgesic effects using chemical-induced (formalin and acetic acid) and heat-induced (tail-immersion and hot plate test) nociception models in mice. The findings were further supported by the possible involvement of opioidergic and GABAergic systems. The findings of the study clearly suggest that *I. dipyrena* is an excellent source of bioactive compounds responsible for analgesic effect. These characteristics can be linked to intrinsic active compounds such as phenolic and flavonoids found in the highest amounts in *I. dipyrena*. However, further investigations are required to isolate the pharmacologically active secondary metabolites responsible for alleviating pain.

## Data Availability

All data generated or analyzed during this study are included in this published article.

## Abbreviations

Crd-Id: crude extract
nhex-Id: *n*-hexane fraction
Chl-Id: chloroform fraction
Et-Id: ethyl acetate fraction
But-Id: butanol fraction
Aq-Id: aqueous fraction
Fr.Chl-Id 46, 47 and 48: subfractions
